# Gestational Hypoxia and Blood-Brain Barrier Permeability: Early Origins of Cerebrovascular Dysfunction Induced by Epigenetic Mechanisms

**DOI:** 10.3389/fphys.2021.717550

**Published:** 2021-08-19

**Authors:** Emilio A. Herrera, Alejandro González-Candia

**Affiliations:** ^1^Laboratory of Vascular Function and Reactivity, Pathophysiology Program, ICBM, Faculty of Medicine, University of Chile, Santiago, Chile; ^2^Institute of Health Sciences, University O'Higgins, Rancagua, Chile

**Keywords:** chronic intrauterine hypoxia, brain endothelial dysfunction, cerebral circulation, fetal growth restriction, BBB permeability

## Abstract

Fetal chronic hypoxia leads to intrauterine growth restriction (IUGR), which is likely to reduce oxygen delivery to the brain and induce long-term neurological impairments. These indicate a modulatory role for oxygen in cerebrovascular development. During intrauterine hypoxia, the fetal circulation suffers marked adaptations in the fetal cardiac output to maintain oxygen and nutrient delivery to vital organs, known as the “*brain-sparing phenotype*.” This is a well-characterized response; however, little is known about the postnatal course and outcomes of this fetal cerebrovascular adaptation. In addition, several neurodevelopmental disorders have their origins during gestation. Still, few studies have focused on how intrauterine fetal hypoxia modulates the normal brain development of the blood-brain barrier (BBB) in the IUGR neonate. The BBB is a cellular structure formed by the neurovascular unit (NVU) and is organized by a monolayer of endothelial and mural cells. The BBB regulates the entry of plasma cells and molecules from the systemic circulation to the brain. A highly selective permeability system achieves this through integral membrane proteins in brain endothelial cells. BBB breakdown and dysfunction in cerebrovascular diseases lead to leakage of blood components into the brain parenchyma, contributing to neurological deficits. The fetal brain circulation is particularly susceptible in IUGR and is proposed to be one of the main pathological processes deriving BBB disruption. In the last decade, several epigenetic mechanisms activated by IU hypoxia have been proposed to regulate the postnatal BBB permeability. However, few mechanistic studies about this topic are available, and little evidence shows controversy. Therefore, in this mini-review, we analyze the BBB permeability-associated epigenetic mechanisms in the brain exposed to chronic intrauterine hypoxia.

## Introduction

Fetal growth restriction (FGR) is a severe condition during pregnancy, where the fetus does not grow according to its potential as a result of an adverse uterine environment (Kingdom and Smith, 2000). Placental insufficiency is the predominant cause of FGR, leading to chronic fetal hypoxemia and intrauterine growth restriction (IUGR) (Kesavan and Devaskar, 2019). In addition, several babies are exposed to chronic hypoxia and IUGR due to pregnancy at a high altitude (Herrera et al., 2015). Intrauterine hypoxia induces an adaptive fetal redistribution of cardiac output, favoring vital organs such as the brain, known as the brain sparing effect (Giussani, 2016). In this scenario, the cerebral and heart circulations vasodilate, with a concomitant pronounced peripheral vasoconstriction (Villas-Bôas et al., 2008; Giussani, 2016). However, brain vasodilation does not ensure normal brain development in growth-restricted fetuses, and the neurodevelopmental outcomes will depend on the timing of hypoxia, the severity of IUGR, and the gestational age at delivery (Padilla et al., 2011; Baschat, 2014). Studies in animal models have demonstrated that gestational chronic hypoxia reduces the neuronal number and vascular and synaptic numbers in the hippocampus, impairing memory function in adult rats (Camm et al., 2021). In addition, IUGR may compromise cerebral vascular homeostasis by increased excitotoxicity, oxidative stress, and neuroinflammation (Miller et al., 2016; Sweeney et al., 2019). In structural terms, IUGR is associated with reduced brain and cortical volume, showing a reduced number of cells, and myelination shortages. These conditions are evidenced by less efficient networks with decreased long-range connections (Miller et al., 2016). Even more, some authors have proposed an association of fetal hypoxia to later neurodegenerative and neuropsychiatric disorders (Faa et al., 2014, 2016).

**Figure 1:**
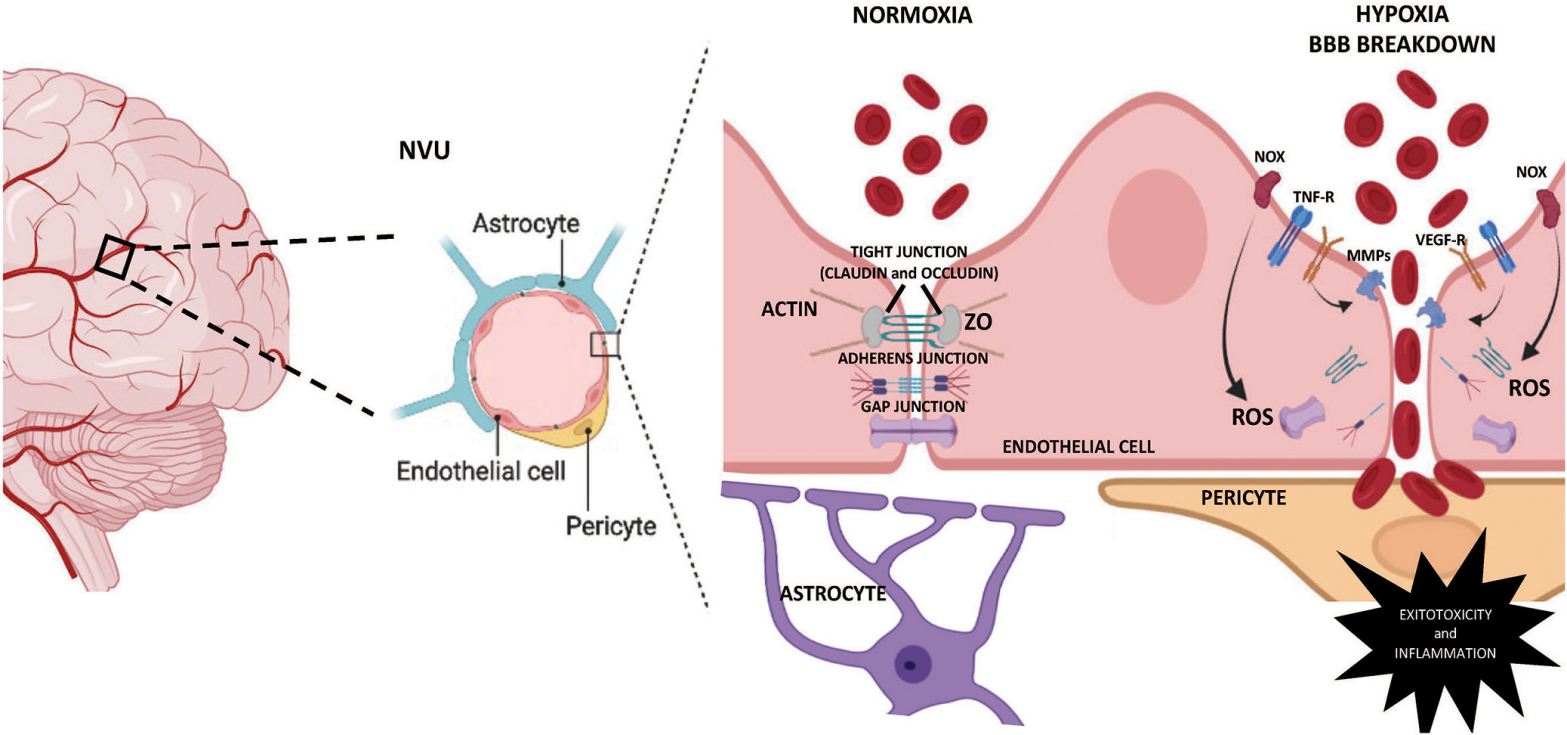
Schematic representation of the BBB permeability mechanisms. The NVU is the main anatomical unit of the blood-brain barrier (BBB), sheltering the brain from systemic influences by limiting transcellular and paracellular transport. The Brain endothelial cells contain no fenestrae and undergo very low rates of transcytosis. Tight junctions, adherens junctions, and gap junctions formed between adjacent endothelial cells underlie the physical barrier that impedes paracellular diffusion of ions, macromolecules, and other solutes. Fetal chronic hypoxia may determine increased permeability in the BBB through pro-inflammatory and oxidative mechanisms, inducing degradation and damage in the membrane's integral proteins, leading to BBB breakdown. MMPs, metalloproteinases; NOx, NADPH oxidase; NVU, neurovascular unit; ROS, reactive oxygen species; TNF-R, Tumor necrosis factor receptor; VEGF-R, vascular endothelial growth factor receptor; ZO, zonula occludens.

However, the principal factor of cerebrovascular diseases is the BBB breakdown, characterized by blood component infiltration, aberrant transport, and clearance of molecules into the central nervous system (CNS) (Yang and Rosenberg, 2011; Zhao et al., 2015). Structurally, the core of the BBB is a monolayer of brain endothelial cells; nevertheless, these cells cannot form a barrier on their own (Gastfriend et al., 2018). Indeed, the development of integrity characteristics in the cerebrovascular tree requires organized cells interactions from glial cells (i.e., astrocytes, microglia), pericytes, and neurons. Such a complex relationship implies the existence of a neurovascular unit (NVU) (Sweeney et al., 2019). The NVU represents a structural and functional multicellular interaction between cerebral parenchyma and brain circulation (Iadecola, 2017), establishing a highly selective BBB that favors cerebral homeostasis (Bell et al., 2020) (Figure 1). The complexity of this unit opens a wide and interesting field in the search for understanding the multiple processes that mediate cerebrovascular health.

## Neurovascular Unit in Intrauterine Growth Restriction

The NVU plays various roles within the brain. This unit is responsible for the homeostasis and regulation of the cerebral blood flow in response to neuronal activity changes, known as neurovascular coupling (NVC) (Iadecola, 2017; Hendrikx et al., 2019). In addition, the same unit is in charge of protecting the CNS from harmful blood-borne and toxic substances (Blanchette and Daneman, 2015; Keaney and Campbell, 2015). From a structural view, three layers determine the barrier function in CNS, (i) the arachnoid barrier, (ii) the blood-cerebrospinal fluid barrier (BCSFB), and (iii) the blood-brain barrier (Tietz and Engelhardt, 2015). While the arachnoid barrier and the BCSFB have moderate permeability in the fetal, neonatal, and adult period, the BBB is the closest structure to the brain cells and hence, is considered the most important barrier (Benz and Liebner, 2020). The BBB is formed by endothelial cells that separate the capillary blood from the brain interstitial fluid and parenchyma, limiting transcellular and paracellular transport mechanisms through a differential expression of tight junctions (TJ), adherens junctions (AJ), and possibly gap junctions (GJ) in the inter-endothelial cleft (Figure 1). Also, the BBB comprises vascular smooth muscle cells, astrocytes, microglia, pericytes, and oligodendrocytes. These cells contribute to the permeability and integrity of the BBB through their intimate anatomical relationship (Liebner et al., 2018). However, the cross-talk between each cell type is partially understood, and our knowledge of neonatal BBB development remains incomplete.

Every constituent cell of the NVU contributes to the BBBs integrity, and any dysfunction might result in the barrier breakdown, with dramatic consequences such as neuroinflammation and neurodegeneration (Kempuraj et al., 2016; Sweeney et al., 2019). Although there is little or no evidence of the effects of IUGR on vascular permeability in human neonates exposed to hypoxia, there is plenty of data obtained from different animal models (Clancy et al., 2001, 2007; Kaur and Ling, 2008; Disdier and Stonestreet, 2020). Thus, the neonatal NVU increases the BBB's permeability by structural changes in the seal given by TJ, AJ, or GJ proteins. Brain endothelial cells contain low fenestration and selective rates of transcytosis mainly due to the high expression of the TJ proteins. TJ are a combination of transmembrane proteins (claudins and occludin) and cytoplasmic adapter proteins called zonula occludens (ZO) that interact with cytoskeleton filaments (Gonzalez-Candia et al., 2021). Decreases in TJ proteins expression have been reported after hypoxic exposition in neonatal brains. Specifically, hypoxia induces a decrease in claudin 5 and occludin protein levels, which increases the paracellular diffusion of solutes and ions across the BBB (Andersson et al., 2021). Furthermore, claudins decrease in the long term is associated with BBB breakdown and neurovascular disorders in humans (Tietz and Engelhardt, 2015). BBB functions have mainly focused on TJs; however, cadherin/catenin interaction, as AJ proteins, regulate cell-cell adhesion between endothelial cells, contributing to the overall junction arrangement and BBB integrity (Li et al., 2018). Vascular endothelial (VE)-cadherin is responsible for the assembly of AJ and is downregulated by BBB breakdown signaling events (Daneman and Prat, 2015). For instance, under neuroinflammatory conditions, PI3Kα triggers TNFα signaling to cause VE-cadherin internalization, reducing the protein levels at junctions and impairing endothelial barrier function (Cain et al., 2010). Gestational or postnatal hypoxia can induce an unbalanced oxidative tone, as described elsewhere (Herrera et al., 2014; Villamor et al., 2019). The induction of the NADPH oxidase (NOX) system by proinflammatory mediators can generate BBB permeability by downregulation of proteins involved in intercellular junctions such as VE-cadherin, occludin, and claudin-5 (Rochfort et al., 2014). Another family of proteins involved in the permeability of the BBB is the GJ, constituted by connexins. Connexin hemichannels have been implicated in the propagation of injury by hypoxia (Kim et al., 2017). Interestingly, neonatal hypoxia can negatively regulate the expression of connexin 43 (Davidson et al., 2013). In addition, the blockade of connexin 43 decreased oligodendrocyte death and recovered oligodendrocyte maturation in preterm fetuses exposed to perinatal asphyxia (Davidson et al., 2014).

Inflammatory mediators are critical for BBB disruption. Microglia, neurons, astrocytes, and endothelial cells can release proinflammatory cytokines and chemokines, modulating adhesion molecules and transmigration of activated immune cells into the brain parenchyma (Jickling et al., 2015; Huang et al., 2016). In endothelial cells, proinflammatory molecules regulate the expression of adhesion molecules such as intercellular adhesion molecule 1 (ICAM-1) and vascular cell adhesion protein 1 (VCAM-1), physiologically expressed at low levels in the BBB. However, their expression is increased in response to hypoxia, increasing the extravasation of molecules into the brain parenchyma (Kong et al., 2018).

In addition, the increase in cellular levels of TNF-α and IL-1β has been related to the decrease in occludin expression and ZO-1 and 2 in the hypoxic brain (Rochfort and Cummins, 2015; Abdullah et al., 2018). This causes an increased paracellular permeability, modulation of transcytosis, and endocytotic transport mechanisms, leading to changes in transcellular transport and inflammatory damage in the brain parenchyma (Sweeney et al., 2019). Besides, reactive glial cells, members of the NVU, are likely to contribute to the permeability of the BBB observed in cerebral hypoxia through downregulation of paracellular proteins such as Claudin-5 (CLDN5), occludin, and ZO-1 (Obermeier et al., 2013). On the other hand, hypoxia-induced vascular endothelial growth factor (VEGF) type 2 receptor (VEGFR-2) pathway activation, increasing permeability in the brain microvascular endothelium by decreasing junctional proteins claudin-5, occludin, and ZO-1 (Castañeda-Cabral et al., 2020). Besides, in postnatal cerebral ischemia, VEGF may affect BBB damage by inducing metalloproteinases (MMP)-2 expression, increasing the BBB permeability by brain endothelial dysfunction (Shen et al., 2018).

Oxidative stress has a critical role in BBB breakdown in different neurological conditions (Olmec and Ozyurt, 2012). Although the hypoxia generated in IUGR is sufficient to generate a redox imbalance (Myatt and Cui, 2004; Herrera et al., 2014), direct evidence in human or animal models of BBB permeability is associated with IUGR remains to be elucidated. CNS contains several sources of ROS, such as NOX, uncoupling of the mitochondrial electron transport chain, xanthine oxidase isoform, and uncoupled nitric oxide synthase (NOS) (Warner et al., 2004). The NOX family seems to be a principal source of oxidative stress in the hypoxic brain through the generation of superoxide (O2•-) radicals (Yang et al., 2019). The predominant isoform is the NOX2 in brain endothelial cells, and it has been observed that the Nox2- knockout mice induce less MMP-9 and diminished expression of occludin, a critical protein of the BBB permeability (Liu et al., 2011). In addition, ROS generated by NOX can act as activators of MMPs (Li et al., 2018), thus enhancing their proteolytic degradation to the BBB. Among MMP family members, MMP-2 and 9 possess a substrate specificity for fibronectin, laminin, collagen fibers, and TJ, all of them structural components of the BBB. Interestingly, these proteins can be induced by hypoxia (Rosenberg and Yang, 2007).

## Perinatal Programming of The NVU: Potential Epigenetic Mechanisms

Adverse environmental conditions during development, such as prenatal hypoxia, can increase the risk of diseases in adulthood, such as vascular and parenchymal brain diseases (Berson et al., 2018). Basic and translational studies have demonstrated that epigenetic programming of gene patterns in response to gestational stress have a critical function in the fetal origins of neurological cells dysfunction (Ducsay et al., 2018). In particular, during gestational hypoxia, the epigenetic programming of genes determines the functional outcome of the genome (Ducsay et al., 2018). Epigenetics as heritable patterns in gene expression which are not associated with DNA sequence alteration (Smith et al., 2016). The epigenetic mechanisms include methylation and/or demethylation of DNA, post-translational modifications of histones, and non-coding RNAs such as microRNAs (Casanello et al., 2016; Ducsay et al., 2018; Zeng and Chen, 2018). Epigenetic events respond to endogenous and exogenous signals, having central roles in regulating appropriate sets of gene expression (Zeng and Chen, 2018). Epigenetic modifications serve as remembrance in early life stages, that can induce long-term changes in gene expression, which may induce disease in later postnatal life (Ducsay et al., 2018).

Hypoxic stress activates multiple epigenetic mechanisms in the fetal brain that increase the vulnerability for neurodevelopment disturbances in adult offspring (Ma et al., 2014; Faa et al., 2016), such as increased vulnerability to ischemic or hypoxic insults (Li et al., 2012; Gonzalez-Rodriguez et al., 2014), disruption of the normal endocrine axis (Wood et al., 2014), and increased risks for adult cardiovascular disease (Ducsay et al., 2018). The mechanisms underlying the effects of chronic fetal hypoxia and IUGR on epigenetic programming of the fetal brain endothelial cells or NVU has not been studied. However, the effects induced by hypoxia and oxidative stress in the fetal brain suggest the involvement of epigenetic mechanisms (Camm et al., 2021).

**Figure 2:**
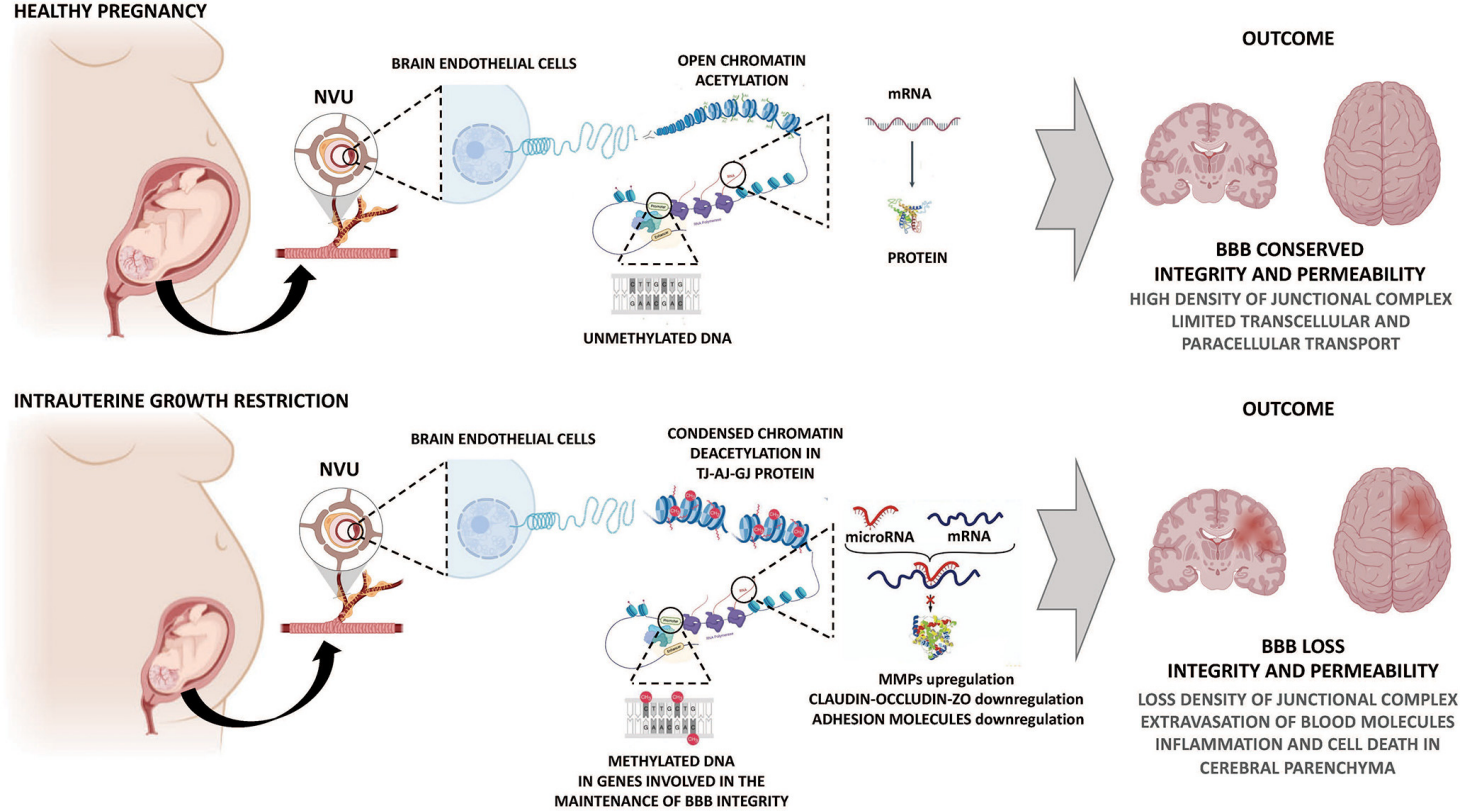
Potential epigenetic mechanisms determining impairment of the BBB permeability by gestational hypoxia. Hypoxic and IUGR injury causes a series of molecular events, including programming of BBB through genomic DNA methylation signature and an altered expression of microRNAs. These mechanisms contribute to changes in the expression of molecules related to junctional complexes of the BBB, increasing BBB permeability and further brain damage. AJ, adherens junction; BBB, blood-brain barrier; GJ, gap junction; MMPs: metalloproteinases; NVU, neurovascular unit; TJ, tight junction; ZO, zonula occludens.

DNA methylation regulates the accessibility of DNA to the transcription machinery modifying the chromatin state. This DNA methylation is generated by a group of enzymes known as DNA methyltransferases (DNMTs) (Ducsay et al., 2018); composed by three principal isoforms: maintenance DNMTs (DNMT1) and *de novo* DNMTs (DNMT3a and DNMT3b) (Moore et al., 2013). However, no mechanisms have been proposed to demonstrate the DNA methylation events during gestational hypoxia, that may regulate the expression of BBB structural proteins and permeability. In this sense, the effects of hypoxia and IUGR can only be extrapolated in neonatal studies or adult models of cerebral ischemia. In models of cerebral hypoxia-ischemia, an increase of DNA methylation was described as an increase in global DNA methylation in the murine cerebral hemispheres, in the promoter of tissue inhibitors of MMP-2 (TIMP2). Increased MMP-2 and MMP-9 expression and activity can affect BBB permeability by proteolysis of extracellular matrix and structural proteins in brain endothelial cells, increasing the BBB breakdown (Figure 2) (Yang et al., 2007; Wang et al., 2012). Late gestational maternal hypoxia in rats induce hypomethylation in the fetal brain by a mechanism dependent on HIF-1α expression (Li et al., 2016). This is relevant as the HIF-related pathway is recognized as the primary sensor and effector for hypoxic cellular adaptation in the fetus (Herrera et al., 2014). Hypomethylation induced by maternal hypoxia increased the vulnerability to subsequent postnatal hypoxia and worsened neurobehavioral outcomes in rat pups (Chen et al., 2008; Li et al., 2016). Interestingly, some authors have shown that HIF-1 expression levels and its transcriptional activity are under strong epigenetic regulation (Nguyen et al., 2013; Ma et al., 2014) and others that HIF-1 itself controls the expression of several epigenetic regulators (Bustelo et al., 2020). The role of HIF in BBB functional programming during fetal hypoxia is still unknown and needs further study.

Histone modification by acetylation and deacetylation plays a central role in chromatin remodeling and epigenetic regulation. In particular, histone deacetylases (HDAC) are potential therapeutic targets in different neurological conditions (Gräff and Tsai, 2013). In a recent study, the treatment with a HDAC inhibitor in mice subjected to cerebral ischemia leads to an enhanced expression of the TJ proteins ZO-1, Occludin, and Claudin-5 in brain endothelial cells, further decreasing the BBB permeability (Su et al., 2020). Conversely, hypoxia and glucose deprivation in the brain promotes HDAC9 expression in endothelial cells, which has been associated to decreased expression of ZO-1, claudin-5, and occludin (Shi et al., 2016). These findings demonstrate the effect of hypoxia on the post-translational modifications of histones in the regulation of proteins involved in the maintenance of the BBB structure and that these mechanisms may be determining the dysfunction of the BBB induced by the hypoxia.

Another mechanism of epigenetic regulation is mediated by microRNAs, which cause the degradation of genes involved in the development and progression of BBB dysfunction (Figure 2) (Ma et al., 2020). Currently, there are no data relating to IUGR and microRNA regulating BBB structure and function; however, evidence in adult pathophysiology may give some clues about microRNAs and BBB disruption. Hypoxic-ischemic models in adult animals have shown that microRNAs can directly or indirectly degrade BBB proteins. In this sense, it has been reported that miR-132 is negatively regulated by hypoxia, which increases MMP-9 activity, which degrades TJ proteins in brain endothelial cells or extracellular matrix components in the NVU, favoring an increased permeability of the BBB (Cichon et al., 2014). There are significant correlations between microRNAs and TJs by hypoxia in adult models (Toyama et al., 2017). For instance, miR-125-5p has a critical role in the brain endothelial tightness during an inflammatory response (Toyama et al., 2017). Part of this response involves specific mRNA targets of miR-125-5p by down-regulating Claudin-1 and Claudin-5, and disrupting adhesion molecules in BBB (Toyama et al., 2017). Furthermore, cerebral endothelial miR-144 downregulates claudin-5, Claudin-12, occludin, and ZO-1, ZO-2, and ZO-3 in a model of BBB permeability associated with a blood-tumor barrier (Cai et al., 2017). Cerebral ischemia triggers an enhanced expression of miR126, which is considered endothelial-specific. miR126 is one of the most studied microRNAs that regulates vascular inflammation. miR126 downregulates the expression of the ICAM-1 and VCAM-1 molecules and controls inflammatory cells extravasation into the brain in BBB dysfunction models (Stamatovic et al., 2016). These data suggest that epigenetic mechanisms define and regulate the vascular responses to pathological stimuli such as chronic hypoxia (Figure 2). However, evidence from fetal exposure to hypoxia leading to epigenetic modifications remains elusive.

## Conclusion

Chronic deprivation of oxygen during gestation dramatically impacts fetal brain development. Gestational hypoxia can act through an altered epigenetic fashion to compromise placental and vascular function (Gheorghe et al., 2010; Herrera et al., 2014; Fajersztajn and Veras, 2017; Soares et al., 2017). However, advances in understanding how gestational hypoxia induces variations in the expression of proteins involved in the integrity of the cerebrovascular network remain widely unexplored. BBB permeability is a major factor determining the cause, progression, outcome, and therapeutic effectiveness of different neurological impairments in postnatal life. Therefore, fetal programming of BBB permeability by hypoxia and IUGR pose a unique challenge to the scientific community in searching for involved mechanisms and effective clinical treatment to prevent detrimental postnatal outcomes.

## Author Contributions

AG-C and EH drafted and edited the manuscript. Both authors contributed to the article and approved the submitted version.

## Conflict of Interest

The authors declare that the research was conducted in the absence of any commercial or financial relationships that could be construed as a potential conflict of interest.

## Publisher's Note

All claims expressed in this article are solely those of the authors and do not necessarily represent those of their affiliated organizations, or those of the publisher, the editors and the reviewers. Any product that may be evaluated in this article, or claim that may be made by its manufacturer, is not guaranteed or endorsed by the publisher.
